# Deficiency of the Complement Component 3 but Not Factor B Aggravates Staphylococcus aureus Septic Arthritis in Mice

**DOI:** 10.1128/IAI.01520-15

**Published:** 2016-03-24

**Authors:** Manli Na, Anders Jarneborn, Abukar Ali, Amanda Welin, Malin Magnusson, Anna Stokowska, Marcela Pekna, Tao Jin

**Affiliations:** a Department of Rheumatology and Inflammation Research, Institution of Medicine, Sahlgrenska Academy at University of Gothenburg, Gothenburg, Sweden; b Center for Brain Repair and Rehabilitation, Department of Clinical Neuroscience and Rehabilitation, Institute of Neuroscience and Physiology, Sahlgrenska Academy at the University of Gothenburg, Gothenburg, Sweden; c Department of Rheumatology, Sahlgrenska University Hospital, Gothenburg, Sweden

## Abstract

The complement system plays an essential role in the innate immune response and protection against bacterial infections. However, detailed knowledge regarding the role of complement in Staphylococcus aureus septic arthritis is still largely missing. In this study, we elucidated the roles of selected complement proteins in S. aureus septic arthritis. Mice lacking the complement component 3 (*C3*^−/−^), complement factor B (*fB*^−/−^), and receptor for C3-derived anaphylatoxin C3a (*C3aR*^−/−^) and wild-type (WT) control mice were intravenously or intra-articularly inoculated with S. aureus strain Newman. The clinical course of septic arthritis, as well as histopathological and radiological changes in joints, was assessed. After intravenous inoculation, arthritis severity and frequency were significantly higher in *C3*^−/−^ mice than in WT controls, whereas *fB*^−/−^ mice displayed intermediate arthritis severity and frequency. This was in accordance with both histopathological and radiological findings. C3, but not fB, deficiency was associated with greater weight loss, more frequent kidney abscesses, and higher bacterial burden in kidneys. S. aureus opsonized with *C3*^−/−^ sera displayed decreased uptake by mouse peritoneal macrophages compared with bacteria opsonized with WT or *fB*^−/−^ sera. C3aR deficiency had no effect on the course of hematogenous S. aureus septic arthritis. We conclude that C3 deficiency increases susceptibility to hematogenous S. aureus septic arthritis and impairs host bacterial clearance, conceivably due to diminished opsonization and phagocytosis of S. aureus.

## INTRODUCTION

Septic arthritis is considered one of the most dangerous joint diseases owing to its rapidly progressive disease character, relatively high mortality, and poor prognosis. Staphylococcus aureus is the most common cause of bacterial arthritis (1, 2). The risk factors for acquiring septic arthritis include increasing age, preexisting joint diseases, and decreased immunocompetence (1). An additional challenge is posed by increasing antibiotic resistance of S. aureus and the spread of highly virulent methicillin-resistant strains in past decades (3).

Nonspecific innate immune responses, including neutrophils (4) and NK cells (5), are generally considered to be protective against septic arthritis, whereas certain cell types, e.g., CD4^+^ T cells of the acquired immune system, potentiate the severity of disease by triggering exaggerated responses (6, 7). The complement system, one of the essential components of the innate immune response, not only participates in recognizing and eliminating invading microorganisms (8), but also enhances the adaptive immune responses (9). Activation of complement by S. aureus can be mediated through all three different pathways, classical, lectin, and alternative, all of which share the common step of activating the central component, complement component 3 (C3), which generates bacterium-bound opsonin, C3b; anaphylatoxins C3a and C5a; and the formation of the lytic membrane attack complex (MAC). Gram-positive bacteria are generally protected from MAC (C5b-9)-mediated lysis by their thick peptidoglycan layer (10). However, the very distinct location of C5b-9 deposits on their cell surfaces, which contrasts with the random deposition of C3b, suggests some yet-to-be-determined function of C5b-9 (11). The role of the complement system was intensively studied in a mouse model of S. aureus sepsis. It has been shown that C3 is more critical than C4 and C5 in controlling S. aureus bacteremia. Also, complement receptor 1 and 2 deficiency led to increased mortality in mice with S. aureus bacteremia (12, 13). Compared to C3, mannose-binding lectin deficiency had a smaller but significant effect on survival of S. aureus sepsis, and this effect was not C3 dependent (14, 15). So far, however, very little is known about the specific role of the complement system in the pathogenesis of septic arthritis. The only study was done by Sakiniene et al. using cobra venom factor to induce an enormous activation of the complement system, resulting in complement depletion. Complement depletion by this strategy significantly aggravated S. aureus septic arthritis in mice (16). However, this strategy does not allow the elucidation of the exact roles of different complement proteins in S. aureus septic arthritis.

In the present study, we compared the susceptibilities to S. aureus septic arthritis of mice lacking C3, complement factor B (fB), and receptor for C3-derived anaphylatoxin C3a (C3aR) using our well-established murine models for S. aureus arthritis. Our data demonstrate that C3 deficiency greatly increased susceptibility to staphylococcal hematogenous septic arthritis. In contrast, neither C3aR nor fB deficiency had a significant effect on the development of septic arthritis.

## MATERIALS AND METHODS

### Mice.

*C3*^−/−^ (17), *C3aR*^−/−^ (18), and *fB*^−/−^ (19) mice were backcrossed to the C57BL/6 genetic background for 10 generations. The mice were kept under standard conditions of temperature and light and were fed laboratory chow and water *ad libitum*. Mice of both sexes were used for experiments at the age of 6 to 10 weeks. For each experiment, the ages and sexes of the mice were matched. The ethics committee of animal research of Gothenburg approved the study.

### Bacterial strain and reagents.

S. aureus strain Newman was cultured on blood agar plates for 24 h, harvested, and kept frozen at −20°C in phosphate-buffered saline (PBS) containing 5% bovine serum albumin (BSA) and 10% dimethyl sulfoxide (DMSO). Before experiments, the bacterial suspension was thawed, washed in PBS, and adjusted to the required concentration (20).

### Mouse model for hematogenous S. aureus arthritis.

We used a well-established mouse model of septic arthritis closely resembling the human infectious arthritis that is hematogenously spread (21). Mice were inoculated intravenously (i.v.) in the tail vein with 0.2 ml of staphylococcal suspension and euthanized on day 10 postinoculation (22).

First, we sought to find the optimal arthritogenic dose for *C3*^−/−^ mice. Different doses (1 × 10^5^ to 1 × 10^7^ CFU/mouse) of S. aureus Newman were used. As a dose of 4 × 10^6^ CFU/mouse induced septic arthritis in around 65% of *C3*^−/−^ animals, this dose was chosen for all other experiments. To study the roles of C3, C3aR, and factor B in hematogenous staphylococcal arthritis, all the mice (*C3*^−/−^, *fB*^−/−^, *C3aR*^−/−^, and wild type [WT]; *n* = 10 to 29) were intravenously inoculated with 4 × 10^6^ CFU of S. aureus Newman. The mice were regularly weighed and examined for arthritis by observers blinded to the groups (T.J. and A.A.). On day 10, the mice were killed, kidneys were obtained for assessment of kidney abscesses and bacterial persistence, serum samples were collected to assess cytokine levels, and paws were obtained for radiological examination of bone erosions followed by microscopic evaluation of synovitis and destruction of cartilage and bone.

To study whether opsonization of S. aureus in WT serum prior to inoculation would affect the clinical course of hematogenous septic arthritis in *C3*^−/−^ mice, S. aureus Newman bacteria were incubated with 25% sera from WT and *C3*^−/−^ mice in PBS at 37°C for 30 min. Incubation with mouse sera did not influence the bacterial viable counts (data not shown). The bacteria were then diluted to the expected concentration and injected i.v. into *C3*^−/−^ mice (*n* = 10/group). The mice were regularly followed by observers blinded to the treatment groups (T.J. and A.A.) for 10 days, and kidneys were obtained for assessment of kidney abscesses.

### Clinical evaluation of arthritis.

Observers blinded to the treatment groups visually inspected all 4 limbs of each mouse (23). Arthritis was defined as erythema and/or swelling of the joints. To evaluate the severity of arthritis, a clinical scoring system ranging from 0 to 3 was used for each paw (0, no inflammation; 1, mild visible swelling and/or erythema; 2, moderate swelling and/or erythema; 3, marked swelling and/or erythema). The arthritis index was constructed by adding the scores from all 4 limbs for each animal as described previously (23, 24). Arthritis that involved 2 or more joints simultaneously was defined as polyarthritis. Since the signs of septic arthritis in deeper joints (e.g., the knee and elbow joints) are impossible to evaluate clinically, micro-computed tomography (μCT) and histopathological examination of joints were further used to confirm the clinical arthritis data.

### Bacteriologic examination.

Kidneys were aseptically removed and blindly assessed by one investigator (T.J.) for abscesses. A scoring system ranging from 0 to 3 was used (0, healthy kidneys; 1, 1 or 2 small abscesses on kidneys without structural changes; 2, more than 2 abscesses but <75% of kidney tissue involved; and 3, large amounts of abscesses with >75% of kidney tissue involved) (22). Afterward, the kidneys were homogenized, diluted serially in PBS, and transferred to agar plates containing 5% horse blood. Bacteria were grown for 24 h and quantified as CFU.

### μCT.

Joints were fixed in 4% formaldehyde for 3 days and then transferred to PBS for 24 h. Afterward, all 4 limbs were scanned and reconstructed into a three-dimensional (3D) structure with a Skyscan1176 micro-CT (Bruker, Antwerp, Belgium) with a voxel size of 35 μm. The scanning was done at 55 kV and 455 mA, with a 0.2-mm aluminum filter. The exposure time was 47 ms. The X-ray projections were obtained at 0.7° intervals with a scanning angular rotation of 180°. The projection images were reconstructed into three-dimensional images using NRECON software (version 1.5.1; Bruker). After reconstruction, the 3D structures of each joint were blindly assessed by 2 observers (T.J. and M.M.) using a scoring system from 0 to 3 (0, healthy joint; 1, mild bone destruction; 2, moderate bone destruction; 3, marked bone destruction) (22, 23, 25).

### Mouse model for local S. aureus arthritis.

*C3*^−/−^, *fB*^−/−^, and WT mice (*n* = 10 to 14) were inoculated in the knee joints with 1 × 10^3^ CFU of S. aureus Newman bacteria/joint in a total volume of 20 μl PBS. Viable-cell counts were performed to determine the number of bacteria injected. The mice were killed 7 days later. Knee joints were collected for μCT scan and histological examination.

### Histopathological examination of joints.

After the scanning, the joints were decalcified, embedded in paraffin, and sectioned with a microtome. Tissue sections were stained with hematoxylin and eosin. All the slides were coded and assessed in a blinded manner by two observers (M.M, and M.N.) with regard to the degree of synovitis and cartilage/bone destruction. The extent of synovitis and cartilage/bone destruction was judged as previously described (22, 24).

### Peritoneal macrophage phagocytosis assay.

An imaging flow-cytometry-based method was employed to analyze the phagocytic capacity of peritoneal macrophages, as previously described (26). Briefly, green fluorescent protein (GFP)-expressing S. aureus (multiplicity of infection [MOI], 5) bacteria were incubated with 25% sera from WT, *C3*^−/−^, and *fB*^−/−^ mice in PBS at 37°C for 30 min. Peritoneal leukocytes from 5 WT mice were collected using peritoneal lavage with 10 ml ice-cold PBS. The mixed peritoneal cells were incubated with opsonized GFP-expressing S. aureus bacteria at 37°C for 1 h. The cells were then placed on ice, and macrophages were stained with allophycocyanin (APC)-eFluor780-conjugated rat anti-mouse F4/80 antibody (eBioscience), followed by immediate analysis on an imaging flow cytometer (ImageStreamX MkII and IDEAS software v.6.0; Merck Millipore, Germany). The internalization wizard in IDEAS was used to determine whether the GFP-positive bacteria had internalized or merely bound the phagocytes. Data are presented as the percentage of macrophages with internalized S. aureus.

### Measurement of cytokine levels.

The cytokine levels in serum were determined using a Cytometric Bead Array (CBA) mouse inflammation cytokine kit (BD Biosciences) and analyzed using a FacsCanto2 flow cytometer (BD Biosciences). The data were analyzed using FCAP array software (BD Biosciences). The levels of monocyte chemoattractant protein 1 (MCP-1), chemokine (C-C motif) ligand 5 (CCL-5), and receptor activator of nuclear factor kappa B ligand (RANKL) in serum were quantified using DuoSet ELISA Development System kits (R&D Systems, Abingdon, United Kingdom) according to the manufacturer's protocols.

### Statistical analysis.

Statistical significance was assessed using the Kruskal-Wallis test, *post hoc* Dunn's multiple-comparison test, or Mann-Whitney test for continuous variables and the chi-square test or Fisher's exact test for categorical variables. The results are reported as the mean and the standard error of the mean (SEM) unless otherwise stated. *P* values of <0.05 were considered statistically significant.

## RESULTS

### C3 deficiency increases the severity and frequency of hematogenous septic arthritis.

In the first experiment, only *C3*^−/−^ and WT mice (*n* = 10/group) were used. *C3*^−/−^ mice developed significantly more severe and frequent clinical arthritis than WT mice (data not shown).

**FIG 1:**
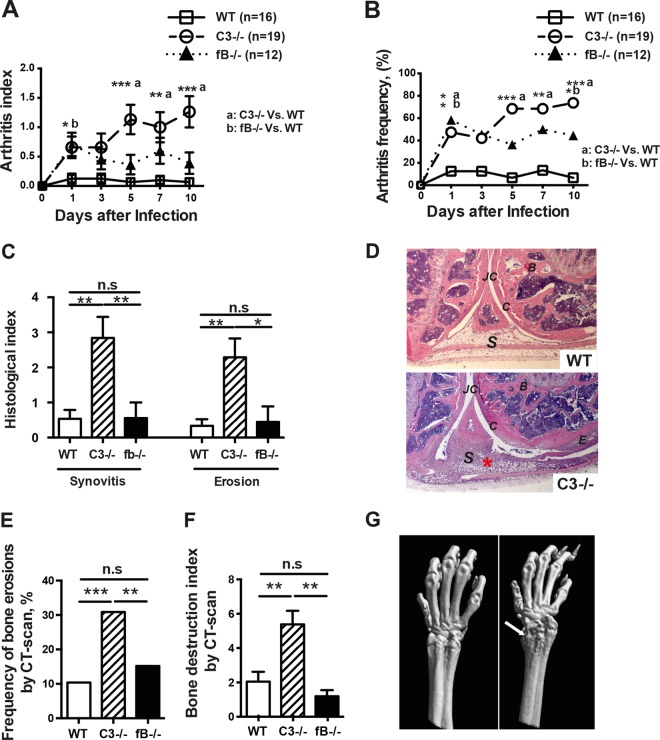
Effects of C3 and fB deficiency on the course of hematogenous staphylococcal arthritis. *C3*^−/−^, *fB*^−/−^, and WT mice were intravenously inoculated with S. aureus strain Newman (4 × 10^6^ CFU/mouse) and sacrificed on day 10. (A and B) The severity (A) and frequency (B) of clinical arthritis were observed for 10 days postinfection. (C) Histological evaluation of the joints from all 4 limbs 10 days after infection. (D) (Top) Micrograph of histologically intact knee joints from a WT mouse inoculated with S. aureus strain Newman. (B) Micrograph of a heavily inflamed knee joint with severe bone and cartilage destruction from a *C3*^−/−^ mouse with septic arthritis (hematoxylin and eosin staining). Original magnification, ×10. The asterisk indicates heavily inflamed synovium. B, bone; C, cartilage; E, erosion of bone and cartilage; JC, joint cavity; S, synovial tissue. (E and F) Frequency of bone destruction (E) and cumulative bone destruction scores (F) of the joints from all 4 limbs of WT, *C3*^−/−^, and *fB*^−/−^ mice as assessed by micro-computed tomography scan. (G) (Left) Intact wrist from a WT mouse inoculated with S. aureus. (Right) Heavily destroyed wrist (both distal radius and ulna) joints from a *C3*^−/−^ mouse with septic arthritis. The arrow indicates bone destruction. The values shown are means and SEM. ***, *P* < 0.001; **, *P* < 0.01; *, *P* < 0.05; n.s, not significant (Kruskal-Wallis test followed by Dunn's multiple-comparison test or chi-square test).

Next, to elucidate the contributions of alternative pathways to the above-mentioned findings, *C3*^−/−^, *fB*^−/−^, and WT mice were simultaneously infected i.v. with S. aureus in the second experiment. Compared to WT mice, *C3*^−/−^ mice developed significantly more severe clinical arthritis as early as day 5, and with time, the difference increased and stabilized until the end of the experiment, whereas *fB*^−/−^ mice had more severe clinical arthritis only on day 1 (Fig. 1A).

The frequency of arthritis was higher in the *C3*^−/−^ mice than in the WT mice (Fig. 1B). Already on day 1, 47% of *C3*^−/−^ mice had developed arthritis versus 12% of WT mice (*P* < 0.05), and at the end of the experiment, the difference was even more pronounced (74% versus 7%; *P* < 0.001) (Fig. 1B). *fB*^−/−^ mice showed an intermediate arthritis frequency (44% on day 10).

Since the arthritis of deeper joints was impossible to assess by clinical evaluation, histopathological examination of all joints was used. In line with clinical arthritis data, histopathologically verified synovitis and the extent of joint destruction were significantly enhanced in *C3*^−/−^ mice compared with WT controls and *fB*^−/−^ mice (Fig. 1C). A joint with septic arthritis on day 10 after infection is typically identified by heavily inflamed synovium and severe cartilage and bone erosions (Fig. 1D, bottom), whereas a healthy joint is characterized by a single-layer synovium without inflammatory infiltration and intact cartilage (Fig. 1D, top). The *fB*^−/−^ and WT mice showed comparable extents of arthritis at the histopathological level.

Importantly, the data from μCT, a state of the art technique for the assessment of joint changes, confirmed the results from histopathological examination. *C3*^−/−^ mice had significantly more joints with bone erosion on the μCT scan than WT (*P* < 0.0001) and *fB*^−/−^ (*P* < 0.01) mice, whereas no difference was observed between *fB*^−/−^ and WT mice (Fig. 1E). A similar pattern was observed with regard to the severity of bone erosion (Fig. 1F).

Next, we performed a more detailed subgroup analysis to investigate which specific joints were affected by S. aureus infection (Table 1). The extent of bone destruction was significantly higher in the shoulders and hips of the *C3*^−/−^ mice than in those of the WT and *fB*^−/−^ mice. Also, *C3*^−/−^ mice exhibited a significantly higher frequency of bone erosions in the front paws, shoulders, hips, knees, and hind paws than both WT and *fB*^−/−^ mice. Strikingly, no bone erosion was found in elbow joints from any group, suggesting that C3 deficiency facilitates bacterial invasion of the majority of joints, but not the elbow joints.

**TABLE 1:** Subgroup analysis of bone destruction in different joint groups by μCT scan^*a*^

Joint group	Severity (mean ± SEM)			Frequency (%)		
	WT	*C3*^−/−^	*fB*^−/−^	WT	*C3*^−/−^	*fB*^−/−^
Front paws	0.77 ± 0.31	1.78 ± 0.37	0.56 ± 0.22	30	58^*b*^^,^^*c*^	28
Elbows	0	0	0	0	0	0
Shoulders	0.20 ± 0.14	1.03 ± 0.20^*c*^^,^^*d*^	0.25 ± 0.14	13	58^*c*^^,^^*d*^	22
Hips	0.03 ± 0.03	0.57 ± 0.21^*b*^^,^^*c*^	0	3	26^*b*^^,^^*c*^	0
Knees	0.17 ± 0.09	0.54 ± 0.15^*c*^	0.06 ± 0.06	10	37^*b*^^,^^*c*^	6
Hind paws	0.88 ± 0.26	1.51 ± 0.33	0.33 ± 0.17	37	47^*c*^	17

a Statistical evaluations were performed using the Kruskal-Wallis test and Fisher's exact test.

b *P* < 0.05 (*C3*^−/−^ versus WT).

c *P* < 0.05 (*C3*^−/−^ versus *fB*^−/−^).

d *P* < 0.001 (*C3*^−/−^ versus WT).

### C3, but not fB, deficiency led to greater body weight loss and impaired bacterial clearance.

**FIG 2:**
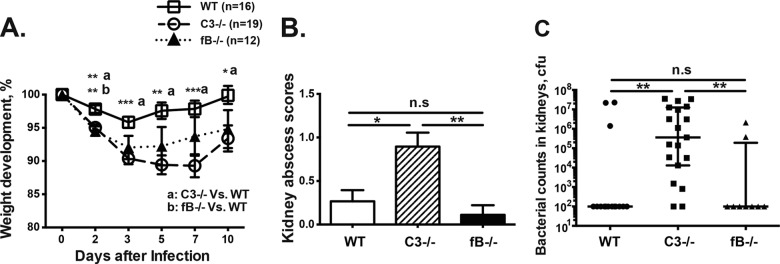
C3 deficiency led to more pronounced body weight loss, more severe kidney abscesses, and higher S. aureus loads in kidneys. WT, *C3*^−/−^, and *fB*^−/−^ mice were intravenously inoculated with S. aureus strain Newman (4 × 10^6^ CFU/mouse), and the animals were sacrificed on day 10. (A) Changes in body weight registered as percentages of the initial body weight. (B and C) Kidney abscess scores (B) and persistence of S. aureus in kidneys 10 days after infection (C). Shown are means and SEM for kidney abscesses and medians with interquartile ranges for the bacterial load in kidneys. ***, *P* < 0.001; **, *P* < 0.01; *, *P* < 0.05; n.s, not significant (Kruskal-Wallis test with Dunn's multiple-comparison test).

Negative body weight development was observed in *C3*^−/−^ mice during the whole course of disease compared with WT mice, whereas *fB*^−/−^ mice had significantly more weight loss than WT mice only on day 1 (Fig. 2A). WT mice lost around 4% of their total body weight by day 3 but started to regain weight thereafter and reached around 99% of their initial body weight by day 10. In contrast, the weight curve of *C3*^−/−^ mice continuously declined to 89% of the initial body weight (*P* < 0.001) on day 7 and slowly recovered to 93% of the initial body weight on day 10 (*P* < 0.05), suggesting more severe infection in *C3*^−/−^ mice.

Macroscopically, more abscesses were observed in the kidneys in *C3*^−/−^ mice than in both WT (*P* < 0.05) and *fB*^−/−^ (*P* < 0.01) mice (Fig. 2B). Good correlation was found between the abscess score and the actual bacterial load in the kidneys (*r* = 0.83; *P* < 0.0001). In line with these results, the *C3*^−/−^ mice had around 3,600-fold higher bacterial load in the kidneys than WT controls (*P* < 0.01) and *fB*^−/−^ mice (*P* < 0.01) (Fig. 2C). This strongly suggests that C3 deficiency impairs the bacterial clearance capacity of the host. In contrast, no differences in the kidney abscess index and kidney bacterial load were found between *fB*^−/−^ and WT mice.

### C3 and fB deficiencies alter serum cytokine profiles in mice with hematogenous S. aureus septic arthritis.

**FIG 3:**
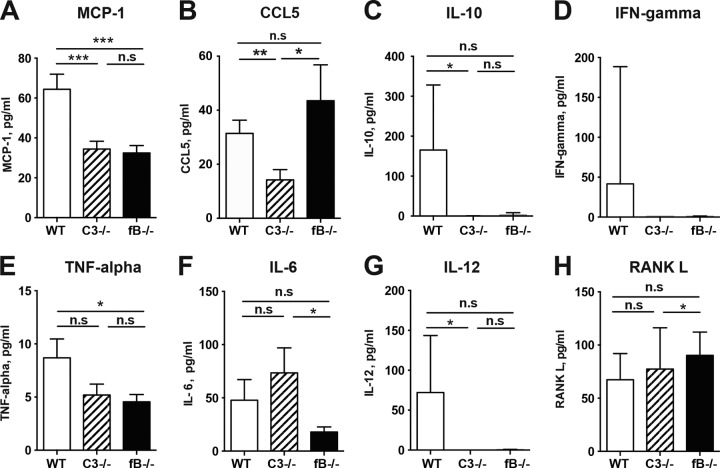
Serum cytokine profiles were altered in C3-deficient and fB-deficient mice inoculated with S. aureus. Levels of five inflammatory cytokines (IFN-γ, TNF-α, IL-6, IL-10, and IL-12), two chemokines (MCP-1 and CCL5), and RANKL in serum were determined after termination of the experiment on day 10 after infection. Shown are means and SEM. ***, *P* < 0.001; **, *P* < 0.01; *, *P* < 0.05; n.s, not significant (Kruskal-Wallis test with *post hoc* Dunn's multiple-comparison test).

To investigate the systemic inflammatory response, we measured the levels in serum of five cytokines (gamma interferon [IFN-γ], tumor necrosis factor alpha [TNF-α], interleukin 6 [IL-6], IL-10, and IL-12), two chemokines (MCP-1 and CCL5), and RANKL (Fig. 3A to G). Significantly lower levels of MCP-1 (*P* < 0.001) (Fig. 3A), CCL5 (*P* < 0.01) (Fig. 3B), IL-10 (*P* < 0.05) (Fig. 3C), and IL-12 (*P* < 0.05) (Fig. 3G) were found in the sera of *C3*^−/−^ mice than in those of WT mice. In contrast, TNF-α (*P* < 0.05) (Fig. 3E) and MCP-1 (*P* < 0.001) (Fig. 3A) levels were lower in *fB*^−/−^ than in WT mice. Intriguingly, significantly higher levels of IL-6 (*P* < 0.05) (Fig. 3F) but lower levels of CCL5 (*P* < 0.05) (Fig. 3B) and RANKL (*P* < 0.05) (Fig. 3H) were found in *C3*^−/−^ than in *fB*^−/−^ mice.

### Peritoneal macrophages displayed impaired phagocytic capacity for S. aureus opsonized with *C3*^−/−^ sera.

**FIG 4:**
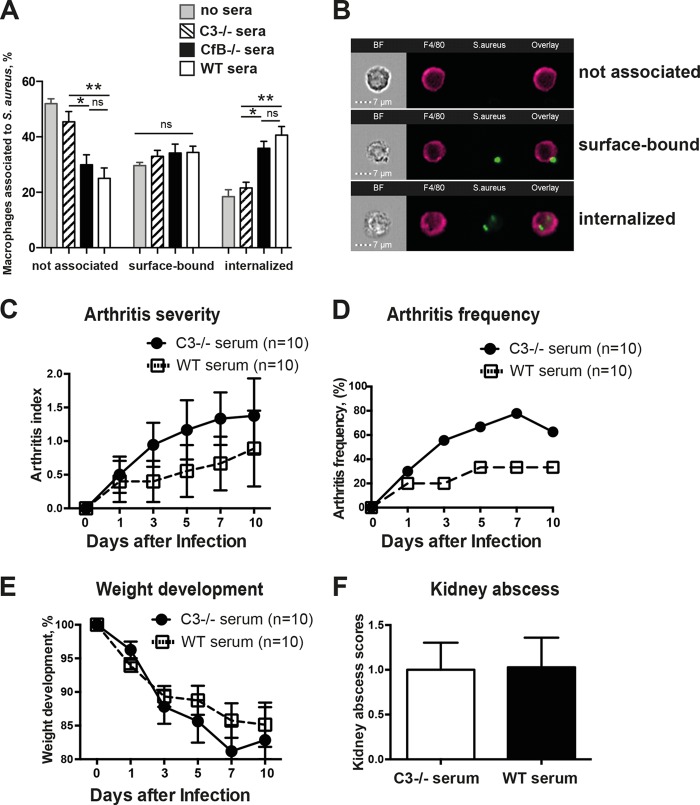
Peritoneal macrophages showed reduced phagocytic capacity for S. aureus opsonized with sera from *C3*^−/−^ mice. Peritoneal leukocytes obtained by peritoneal lavage from five wild-type mice were incubated with GFP-expressing S. aureus (MOI, 5) that was opsonized with sera from WT, *C3*^−/−^, and *fB*^−/−^ mice. The internalization wizard in IDEAS was used to determine the interaction of the GFP-positive bacteria with phagocytes (not associated, surface bound, or internalized). (A) Percentages of macrophages interacting with GFP-positive S. aureus. Shown are means and SEM. **, *P* < 0.01; *, *P* < 0.05; ns, not significant (Kruskal-Wallis test with Dunn's multiple-comparison test). (B) Representative micrographs of macrophages in association with GFP-expressing S. aureus (MOI, 5) analyzed by imaging flow cytometry. S. aureus Newman was incubated with 25% sera from WT and *C3*^−/−^ mice in PBS at 37°C for 30 min and then diluted to the expected concentration and injected i.v. (4 × 10^6^ CFU/mouse) into *C3*^−/−^ mice. (C and D) The severity (C) and frequency (D) of clinical arthritis were observed for 10 days postinfection. (E) Changes in body weight registered as percentages of the initial body weight. (F) Kidney abscess scores 10 days after infection. Shown are means and SEM.

C3b acts as an opsonin that enhances the phagocytic capacity of cells, including neutrophils and macrophages, for bacteria. Image streaming, a state of the art technology, was applied to study the roles of C3 and fB in the phagocytic capacity of macrophages (Fig. 4). Bacterial numbers were similar after 30 min of incubation with *C3*^−/−^, *fB*^−/−^, and WT sera. Also, the phagocytic capacity of peritoneal macrophages from *C3*^−/−^ mice was intact when S. aureus was opsonized with sera from WT mice (data not shown). However, the percentage of macrophages with internalized S. aureus opsonized with *C3*^−/−^ sera was comparable to the internalization frequency of nonopsonized S. aureus (18% versus 21% [not significant]). Importantly, the internalization frequency was significantly increased when S. aureus was opsonized with sera from WT mice (41%; *P* < 0.01) or sera from *fB*^−/−^ mice (36%; *P* < 0.05), suggesting the crucial role of C3, but not fB, in the phagocytic capacity of macrophages for S. aureus.

Next, we asked whether opsonization of S. aureus in WT serum prior to inoculation would affect the clinical course of arthritis after hematogenous inoculation in *C3*^−/−^ mice. Bacteria were incubated with *C3*^−/−^ or WT serum for 30 min, washed, and injected into *C3*^−/−^ mice. We observed a trend toward lower clinical arthritis scores and reduced arthritis frequency in mice inoculated with S. aureus that was preincubated with WT serum (Fig. 4C to F). Taken together, these results show that C3-mediated opsonization of S. aureus is critical for host defense against S. aureus hematogenous septic arthritis.

### C3aR deficiency had no effect on the course of hematogenous septic arthritis.

**FIG 5:**
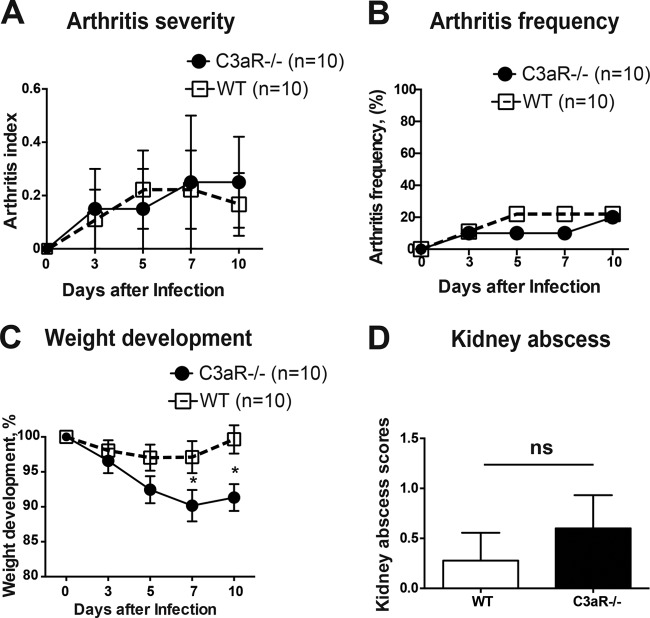
C3aR deficiency did not affect the course of hematogenous staphylococcal arthritis. *C3aR*^−/−^ and WT mice were intravenously inoculated with S. aureus strain Newman (4 × 10^6^ CFU/mouse) and killed on day 10. (A and B) The severity (A) and frequency (B) of clinical arthritis were observed for 10 days postinfection. (C) Changes in body weight registered as percentages of the initial body weight. (D) Kidney abscess scores 10 days after infection. Shown are means and SEM. *, *P* < 0.05; ns, not significant. Mann-Whitney *U* test or Fisher's exact test.

C3a exerts its proinflammatory functions by activating C3aR, whereas C3b functions as an opsonin (27). To elucidate whether the C3a/C3aR interaction plays a role in S. aureus septic arthritis, *C3aR*^−/−^ and WT mice were i.v. inoculated with S. aureus Newman and the course of septic arthritis was followed for 10 days (Fig. 5). No differences were found regarding the severity or frequency of arthritis (Fig. 5A and B). Also, the kidney abscess scores were similar in the two groups (Fig. 5D), although the *C3aR*^−/−^ mice lost more body weight than the WT controls. These results suggest that absence of C3a/C3aR signaling does not contribute to increased susceptibility of *C3*^−/−^ mice to hematogenous septic arthritis.

### Neither C3 nor fB deficiency affects local S. aureus arthritis.

To study the roles of C3 and fB in the late stage of septic arthritis, we injected S. aureus into the knee joints of NMRI (The Naval Medical Research Institute) mice to bypass the early immune responses in the bloodstream. Both synovitis severities and grades of bone erosion were similar among the three groups, which was also confirmed by the μCT scan data (data not shown).

## DISCUSSION

In this study, we demonstrated that C3 deficiency significantly increased both the frequency and severity of S. aureus hematogenous septic arthritis in mice. C3-deficient mice had impaired ability to clear S. aureus and showed more pronounced weight loss. C3aR deficiency had no impact on the development of hematogenous septic arthritis, suggesting that the observed effects of C3 deficiency in hematogenous septic arthritis are largely due to impaired opsonization mediated by C3b rather than C3a-induced proinflammatory responses. In further support of this notion, serum from *C3*^−/−^ mice failed to opsonize the bacteria, and preincubation of S. aureus with WT serum prior to hematogenous injection was associated with a trend toward milder arthritis in *C3*^−/−^ mice. Taken together, these results show that C3-mediated opsonization plays a critical role in protecting the host against S. aureus septic arthritis.

As the great majority of septic arthritis in humans is spread hematogenously, our hematogenous model of septic arthritis induced by intravenous inoculation of S. aureus closely resembles the human disease (21). We propose that the development of hematogenous septic arthritis can be divided into two stages—early and late. During the early stage, S. aureus needs to adapt to the host environment, to survive the bactericidal components and phagocyte attacks in the blood, to disseminate to synovial tissue, and finally to reach the joint cavity. In the joint cavity (late stage), S. aureus proliferates and releases a vast arsenal of components that arouse a host response and cause joint damage. As the intra-articular route of bacterial inoculation bypasses the early stage of disease pathogenesis, our local S. aureus arthritis model can be considered a means to study the later stage of immune responses *in situ*. Our finding that C3 deficiency led to significantly increased arthritis severity in a hematogenous S. aureus arthritis model but not in a local S. aureus arthritis model strongly suggests that the deleterious effect of C3 deficiency in hematogenous septic arthritis was due to the critical role of C3 in the early stage of the disease, i.e., before the bacteria reach the joint cavity.

It takes 2 to 3 days for the signs of clinical septic arthritis to become evident after intravenous inoculation of S. aureus (22, 28), indicating that the early stage of the disease is relatively short. During this short time, neutrophils are the predominant phagocytes responsible for the elimination of invading S. aureus by phagocytosis. Septic arthritis in neutrophil-depleted mice and in C3-deficient mice shows very similar disease patterns, e.g., a higher bacterial load in the kidneys, more severe arthritis, and greater body weight loss (4), indicating a strong connection between neutrophils and C3 in hematogenous septic arthritis.

In septic arthritis, the only initial cause of clinical synovitis and bone erosions is the invading S. aureus in affected joints. Significantly higher frequency of clinical synovitis and CT-verified bone damage in C3-deficient mice strongly suggest that the absence of C3 leads to impaired bacterial clearance and a larger number of S. aureus bacteria reaching more joints. C3a liberated in the course of complement activation has been shown to exhibit antimicrobial activity (29). A direct killing effect of C3a on S. aureus was also observed (30). Direct antimicrobial activity of C3a at the infection site might contribute to host innate immunity, controlling invading pathogens. C3b, another product of C3 activation, is recognized by complement receptors expressed on the phagocytes. This recognition leads to subsequent internalization of the opsonized bacteria by phagocytic cells. The C3b opsonin can be generated by the classical complement pathway, typically initiated by binding of antigen-specific antibody that attracts complement C1q and the consequent formation of the C3 convertase C4b2a. The same C3 convertase is formed in response to carbohydrate structures present on microbial surfaces and is initiated through multimolecular fluid phase complexes composed of a carbohydrate recognition subcomponent (mannose-binding lectin or ficolin L, H, or M) and the lectin pathway serine protease, mannan-binding lectin-associated serine protease 2. In contrast, the alternative pathway of complement activation and the generation of the alternative-pathway C3 convertase (C3bBb) is the result of direct binding of C3b or C3(H_2_O) to the target surface. All three activation pathways have been shown to contribute to the opsonization of S. aureus (31–34). Our data support the conclusion that complement activation by the classical and/or lectin pathway is of critical importance for the clearance of S. aureus from the systemic circulation, whereas the alternative pathway plays only a secondary role in the opsonization of S. aureus used in our study.

In contrast to C3 deficiency, *fB*^−/−^ mice maintained normal production of CCL5, a potent chemokine that recruits neutrophils to the infected tissue via C-C chemokine receptor type 1. Further, fB deficiency had no effect on phagocytosis and was not associated with increased susceptibility to hematogenous septic arthritis. These data further strengthen our conclusion that the alternative pathway of complement activation is not essential for host resistance to S. aureus and the pathogenesis of septic arthritis.

The production of specific antibodies against S. aureus could be impaired in C3-deficient mice, since the complement system is known to maintain the proper function of B cells and mice deficient in complement receptors and C3 have impaired antibody responses to T-cell-dependent antigen (35, 36). Given the short time between inoculation and symptoms of arthritis in our model, our *in vivo* findings and the reduced *in vitro* phagocytosis of bacteria incubated with C3-deficient but not fB-deficient serum could be at least partly due to lower levels of specific antibodies against S. aureus present *a priori* in the blood of mice and not the specific antibody response triggered by the inoculation as such. We have previously shown that both *C3*^−/−^ and *fB*^−/−^ mice respond by low specific antibody production to single immunization with collagen II but that this impaired antibody response is overcome by repeated immunizations (37). Although we have not been able to quantify the specific antibody levels in our mice, it is conceivable that all the mice had had previous and repeated exposure to S. aureus. Hence, the differences between the *C3*^−/−^ and the *fB*^−/−^ and WT mice in clinical arthritis and the opsonization capacity of *C3*^−/−^ versus *fB*^−/−^ and WT sera are conceivably due to the absence of C3 and not due to lower levels of antibodies specific for S. aureus.

As C3a was shown to activate divergent signaling pathways that induce chemokine production (38, 39), it is not surprising that serum MCP-1 and CCL5 levels were significantly lower in C3-deficient mice. Our findings of lower serum IL-12 (a proinflammatory Th1 cytokine) and IL-10 (an anti-inflammatory Th2 cytokine) levels in *C3*^−/−^ than in WT mice also point to the impaired immune responses to S. aureus infection in the absence of C3. RANKL plays a role in bone metabolism and bone resorption (40). However, as RANKL is highly expressed by many cell types, including activated T cells (41), and T-cell responses are reduced in C3-deficient mice, it is not surprising that serum RANKL levels were not significantly elevated in C3-deficient mice despite significantly more severe bone destruction. Our data also suggest that systemic RANKL levels may not be a good indicator of bone destructions in septic arthritis.

It has been suggested that the severity of S. aureus septic arthritis is not exclusively linked to the number of bacteria present in the joint but is also determined by other factors, including proinflammatory cytokines and the extent of leukocyte infiltration in the synovium (22). CD4^+^ T cells are known to be pathogenic in the course of S. aureus arthritis in mice, and pretreatment with anti-CD4 antibodies attenuated the severity of S. aureus septic arthritis in mice (6, 7). The complement system is known to regulate T-cell responses (42). In C3-deficient mice, the priming of both CD4^+^ and CD8^+^ T cells was impaired in an influenza virus model (43). Intracellular C3 convertase-independent C3a generation and C3aR activation contribute to homeostatic mTOR activity and T-cell survival, and increased intracellular C3 activation underlies T effector dysregulation in arthritis (44). Direct signaling by C3a and C5a through their receptors on lung dendritic cells is required for their efficient trafficking to the draining lymph nodes, which is a crucial step for the initiation of T-cell responses (45). Upon T-cell activation, both C3aR and C5aR expression is induced on T cells, and these cells respond to C3a and C5a with directed chemotactic migration (46, 47). Our findings that C3aR deficiency did not alter arthritis severity in mice with hematogenous septic arthritis, however, strongly support the conclusion that, compared to the opsonization effect of C3b, the involvement of C3a/C3aR-mediated proinflammatory processes plays only a minor role in the development of septic arthritis.

Due to widespread and excessive consumption of antibiotics, antibiotic resistance in S. aureus has expanded worldwide and continues to expand at an accelerating rate. There is an urgent need for novel treatment strategies, including improvement of innate immune defense to clear human pathogens, as well as the discovery of new antibiotics. Our data demonstrate the essential protective role of C3-mediated opsonization in host defense against S. aureus hematogenous septic arthritis and raise the possibility of enhancing C3 function for new antistaphylococcal treatments. S. aureus uses a range of strategies to escape complement attack, e.g., by producing complement-inhibitory proteins that act on C3 or C3 convertases, such as extracellular fibrinogen-binding protein (48, 49) and staphylococcal complement inhibitor (SCIN) (50). Neutralization of these key staphylococcal virulence mechanism by vaccination or small molecules blocking the interaction of inhibitory proteins with C3, therefore, represents an attractive therapeutic approach for S. aureus septic arthritis.
